# Improving the Performance of Ultrathin ZnO TFTs Using High-Pressure Hydrogen Annealing

**DOI:** 10.3390/nano15191484

**Published:** 2025-09-28

**Authors:** Hae-Won Lee, Minjae Kim, Jae Hyeon Jun, Useok Choi, Byoung Hun Lee

**Affiliations:** 1 Center for Semiconductor Technology Convergence, Department of Electrical Engineering, Pohang University of Science and Technology, Cheongam-ro 77, Nam-gu, Pohang 37673, Gyeongbuk, Republic of Korea; haewon2612@postech.ac.kr (H.-W.L.); mjkim11@postech.ac.kr (M.K.); jhjun@postech.ac.kr (J.H.J.); 2 Graduate School of Semiconductor Technology, Pohang University of Science and Technology, Cheongam-ro 77, Nam-gu, Pohang 37673, Gyeongbuk, Republic of Korea; useok991122@postech.ac.kr

**Keywords:** ultrathin TFTs, ZnO, high-pressure hydrogen annealing, low-frequency noise

## Abstract

Ultrathin oxide semiconductors are promising channel materials for next-generation thin-film transistors (TFTs), but their performance is severely limited by bulk and interface defects as the channel thickness approaches a few nanometers. In this study, we show that high-pressure hydrogen annealing (HPHA) effectively mitigates these limitations in 3.6 nm thick ZnO TFTs. HPHA-treated devices exhibit a nearly four-fold increase in on-current, a steeper subthreshold swing, and a negative shift in threshold voltage compared to reference groups. X-ray photoelectron spectroscopy reveals a marked reduction in oxygen vacancies and hydroxyl groups, while capacitance–voltage measurements confirm more than a three-fold decrease in interface trap density. Low-frequency noise analysis further demonstrates noise suppression and a transition in the dominant noise mechanism from carrier number fluctuation to mobility fluctuation. These results establish HPHA as a robust strategy for defect passivation in ultrathin oxide semiconductor channels and provide critical insights for their integration into future low-power, high-density electronic systems.

## 1. Introduction

Oxide semiconductors such as zinc oxide (ZnO), indium oxide (In_2_O_3_), and indium–gallium–zinc oxide (IGZO) have emerged as a versatile platform for thin-film electronics because of their large band gap (>3 eV), intrinsically high electron mobility, optical transparency, and compatibility with low-temperature and large-area deposition. These attributes have enabled rapid progress in logic circuits, memory, and sensors, positioning oxide semiconductor thin-film transistors (TFTs) as essential building blocks for next-generation electronics [1,2,3,4].

Recently, channel thickness scaling in TFTs has become critical for emerging electronic applications such as monolithic three-dimensional (M3D) integration. As the thickness of the channel layer is scaled below 10 nm, ultrathin oxide semiconductor channels begin to exhibit distinctly different electrostatic and transport characteristics such as enhanced gate control, suppressed short-channel effects, and reduced parasitic capacitance [5,6].

However, aggressive scaling also introduces significant technical challenges. As the channel thickness approaches only a few nanometers, device performance becomes increasingly susceptible to defect-mediated degradation mechanisms. Both bulk traps—such as oxygen vacancies within the film and interface states at the dielectric/semiconductor boundary—contribute to carrier scattering, threshold voltage instability, and higher low-frequency noise [7,8,9,10].

To mitigate these thickness scaling-induced degradations, effective passivation strategies targeting both bulk and interface traps are essential. Conventional thermal annealing can partially reduce traps, but is often insufficient for ultrathin oxide semiconductors where the defect-to-volume ratio is extremely high. In contrast, high-pressure hydrogen annealing (HPHA) enhances the solubility and chemical reactivity of hydrogen, thereby promoting deeper diffusion into the oxide film and more efficient passivation of oxygen vacancies and dangling bonds [11,12,13]. Previous studies have reported that high-pressure annealing significantly reduces defect densities in wide-bandgap oxides and improves device stability [14,15,16,17], suggesting HPHA as a particularly robust approach for ultrathin oxide channels.

In this work, we systematically investigate the impacts of HPHA on 3.6 nm thick ZnO TFTs by comparing devices before annealing, after vacuum annealing, and after HPHA. HPHA-treated devices exhibit enhanced on-current, reduced subthreshold swing, and a negative shift in threshold voltage compared with untreated counterparts. X-ray photoelectron spectroscopy (XPS) reveals the hydrogen-induced modulation of oxygen vacancy concentrations. Furthermore, the interface trap density, (D_it_) extracted from capacitance–voltage measurements, decreases by more than three-fold. Interestingly, we found that the dominant noise generation mechanism is changed from carrier number fluctuation (McWhorter model) to mobility fluctuation (Hooge model), confirming effective trap passivation by HPHA.

## 2. Materials and Methods

**Figure 1:**
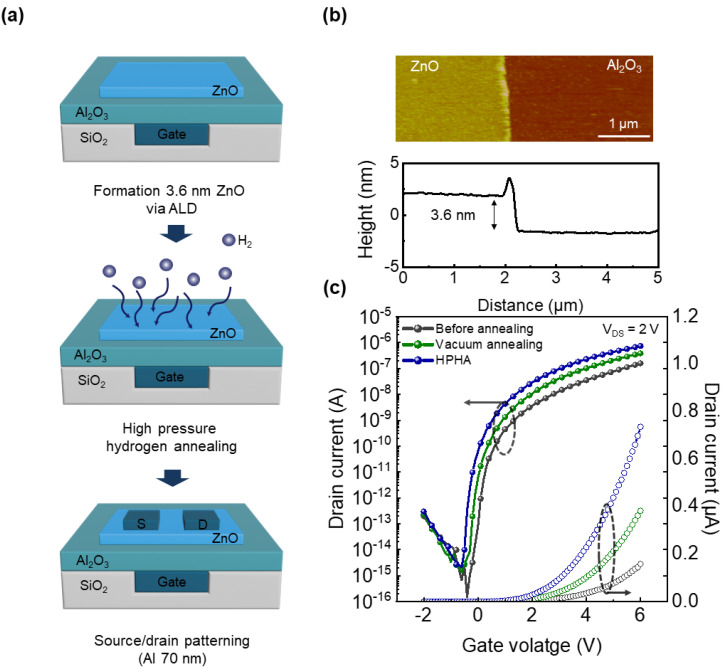
(**a**) Three-dimensional schematic of the fabrication process flow for ultrathin ZnO TFTs incorporating high-pressure hydrogen annealing (HPHA). (**b**) Atomic force microscopy (AFM) image and thickness profile of the ZnO film (3.6 nm), confirming smooth surface morphology. (**c**) Transfer characteristics of ZnO TFTs before annealing, after vacuum annealing, and after HPHA, highlighting the significant performance improvement induced by HPHA.

Figure 1a illustrates the fabrication process of the ultrathin ZnO TFTs, incorporating HPHA. To ensure a uniform electric field distribution in the channel region, a buried gate structure was used [18]. The SiO_2_ trench pattern was formed by reactive ion etching (RIE) with an Ar/CF_4_ mixture. The trench was filled with a 10 nm Ti/60 nm Al metal stack, where Ti served as an adhesion layer and Al as a low-work function favorable for n-type device operation. After planarization of the metal electrode, an 11 nm Al_2_O_3_ layer was subsequently deposited as the gate dielectric, using the atomic layer deposition (ALD) process with trimethylaluminum (TMA) and H_2_O at 200 °C. A 3.6 nm ZnO channel layer was then deposited by ALD with diethylzinc (DEZ) and H_2_O precursors at 100 °C to form the n-type channel layer and patterned using photolithography followed by wet etching using 0.68 wt.% HCl for 2 s at 25 °C. Then, HPHA was performed at 300 °C under 10 bar H_2_ for 30 min on the patterned ZnO. After the HPHA, the 70 nm Al source and drain electrodes were deposited and patterned. The fabricated device features channel dimensions of width (W) = 16 μm and length (L) = 12 μm. For comparison, devices were also fabricated with different annealing conditions: without annealing and vacuum annealing at 300 °C for 30 min under ~10^−2^ Torr.

Film thickness and surface roughness were characterized using atomic force microscopy (AFM, Jupiter XR, Oxford Instruments, Abingdon, UK) as shown in Figure 1b. The root mean square surface roughness (R_q_) was measured to be 0.453 nm. As surface roughness is a critical factor in ultrathin thickness, this film maintains a smooth surface [19].

The chemical states of the ZnO films were analyzed using X-ray photoelectron spectroscopy (XPS). The calibration was performed using the 284.8 eV peak in the C 1s spectrum as a reference.

Electrical characteristics were measured using a semiconductor parameter analyzer (Keithley 4200-SCS, Tektronix, Beaverton, OR, USA). The I–V and C–V characteristics were measured at 25 °C under ambient pressure. The MOS capacitor with an Al (bottom)/Al_2_O_3_ (11 nm)/ZnO (3.6 nm)/Al (top) structure with a circular pattern Al top electrode was used to measure C–V. For low-frequency (1/f) noise measurements, the drain current was amplified with a low-noise current preamplifier (SR570, Stanford Research Systems, Sunnyvale, CA, USA) and analyzed using a dynamic signal analyzer (35670A, Agilent, Santa Clara, CA, USA) with a Fast Fourier Transform (FFT) to obtain the drain current noise spectral density.

## 3. Results and Discussion

The electrical characteristics of ZnO TFTs are presented in Figure 1c. The device exhibits a relatively low on-current before annealing. This degradation is primarily attributed to bulk oxygen vacancies and interface traps in the ultrathin ZnO channel. Oxygen vacancies not only generate free carriers but also act as bulk traps; thus, in ultrathin channels with a high trap-to-volume ratio, device performance is strongly degraded by these defects.

After thermal annealing in a vacuum, the on-current increased by two-fold and exhibited a reduced subthreshold swing compared to the unannealed device. This improvement is likely associated with the desorption of residual contaminants such as hydroxyl groups in the film and oxygen rearrangement at the channel/dielectric interface by thermal energy. These effects collectively reduce traps and scattering centers, thereby improving carrier transport [20,21,22,23,24,25].

In contrast, the HPHA-treated devices exhibit a nearly four-fold increase in on-current, along with a steeper subthreshold swing and a negative shift in threshold voltage compared to unannealed devices. The threshold voltage was obtained from the constant-current method, defined as the gate voltage corresponding to a drain current of 10^−10^ A in the linear region of the log–linear I–V curve. The pronounced improvement highlights the critical role of hydrogen in effectively passivating oxygen vacancies and stabilizing the ultrathin ZnO channel. For quantitative comparison, the key device parameters are summarized in Table 1. The effective mobility was calculated using the following equation:

(1) $${µ}_{\mathrm{eff}}\;=\;\frac{g_{d}}{Q_{n}}\frac{L}{W}$$

where Q_n_ is the sheet charge density, g_d_ is the drain conductance, L is the channel length, and W is the width of channel.

**Figure 2:**
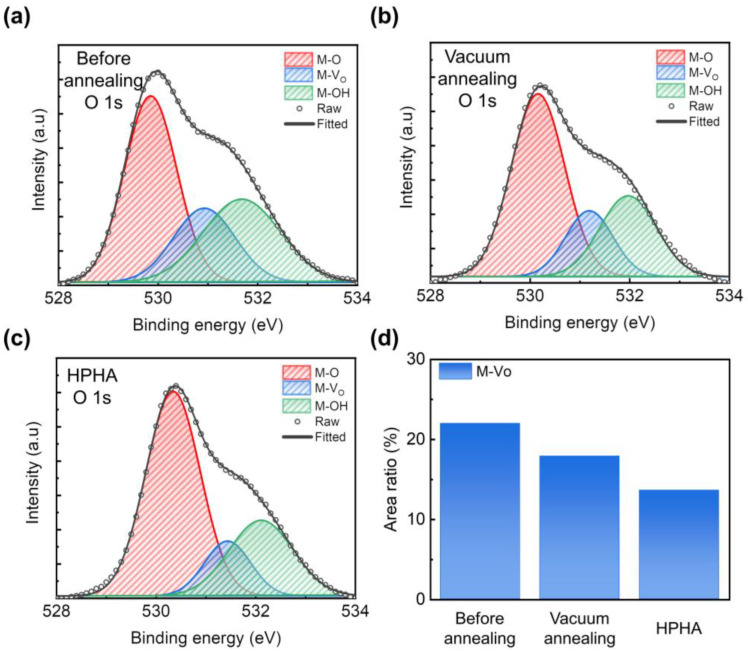
O 1s XPS spectra of ZnO films (**a**) before annealing, (**b**) after vacuum annealing, and (**c**) after high-pressure hydrogen annealing (HPHA), deconvoluted into lattice oxygen (M–O), oxygen vacancies (M–V_O_), and hydroxyl groups (M–OH). (**d**) Relative area ratio of the M–V_O_ component in the O 1s spectra, highlighting the significant reduction in oxygen vacancy density after HPHA.

To elucidate the origin of the performance enhancement observed in HPHA-treated ZnO TFTs, XPS was performed on the three types of ZnO films. Figure 2a–c shows the O 1s core-level spectra deconvoluted into three distinct components corresponding to M–O, M–V_O_, and M–OH. The M–O peak arises from metal–oxygen lattice bonding, while the M–Vo peak reflects oxygen vacancy states caused by missing oxygen atoms at lattice sites. The M–OH peak is assigned to surface hydroxyl groups formed through chemisorbed –OH species or adsorbed H_2_O/O_2_ molecules. These components are centered at binding energies of approximately 530 eV (M–O), 531 eV (M–V_O_), and 532 eV (M–OH), respectively [26].

In the before annealing sample, the O 1s spectrum shows a relatively low M–O contribution (47.2%) and significant fractions of M–V_O_ (21.9%) and M–OH (30.9%), indicating a high density of oxygen-related defects and hydroxyl groups (Figure 2a). After thermal annealing in a vacuum, the M–O fraction increases to 55.8%, accompanied by decreases in M–V_O_ (17.9%) and M–OH (26.3%) (Figure 2b).

The HPHA-treated ZnO film exhibits a pronounced increase in the M–O component (60.1%) along with significant reductions in M–V_O_ (13.6%) and M–OH (26.3%) (Figure 2c). These results demonstrate that hydrogen directly influenced the concentration of oxygen vacancies while also reconstructing the bonding environment toward a more ordered Zn–O network. The progressive oxygen vacancy reduction summarized in Figure 2d indicates that HPHA strongly affects the carrier transport behaviors in the ultrathin ZnO channel region.

**Figure 3:**
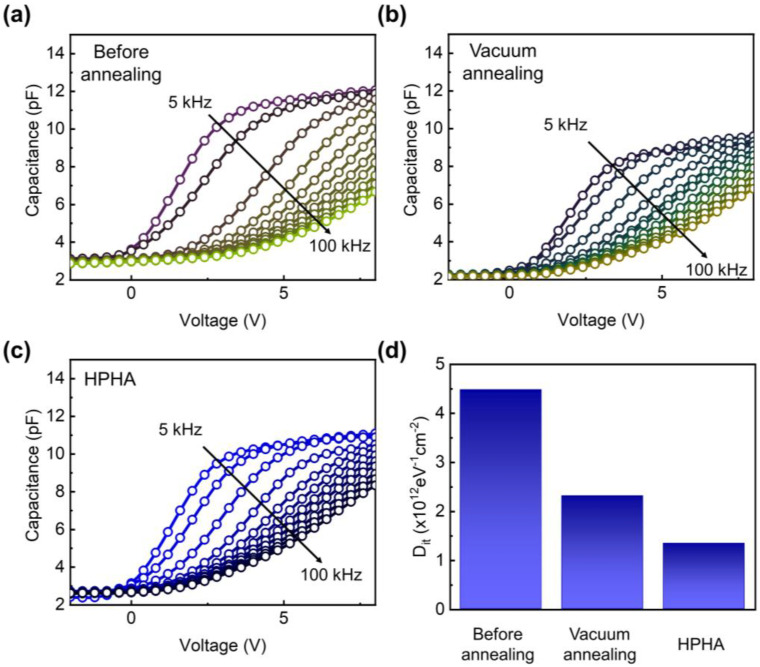
Capacitance–voltage (C–V) characteristics of MOS structure measured at frequencies ranging from 5 kHz to 100 kHz for (**a**) before annealing, (**b**) after annealing in vacuum, and (**c**) after HPHA. (**d**) Extracted interface trap density (D_it_) values obtained using the high–low-frequency method at V_TH_, showing a progressive reduction after annealing and a pronounced decrease following HPHA.

To investigate the impacts of HPHA, charge trapping behaviors in the ZnO channel region should be directly examined. Unfortunately, the characterization of charge trapping and de-trapping in the ZnO region is not as straightforward as in the bulk silicon MOSFETs because it is difficult to use a typical analysis method such as charge pumping analysis, which requires a body contact. To overcome this limitation, we used capacitance–voltage (C–V) measurements performed over a frequency range of 5 kHz and 10–100 kHz in 10 kHz steps with an amplitude of 300 mV. Figure 3a–c shows the capacitance dispersion as a function of frequency. Dispersion near depletion is primarily attributed to interface traps, whereas the dispersion in the accumulation regime is governed by border traps [27].

As shown in Figure 3a, the unannealed device exhibits pronounced frequency dispersion around V_TH_, indicating the presence of a high density of interface traps (D_it_). The frequency dependence arises because traps and carriers respond differently to the AC signal. As D_it_ decreases, the trap-induced capacitance at low frequencies is suppressed, leading to a reduction in frequency dispersion. Both vacuum-annealed and HPHA-treated devices show significantly reduced frequency dependence around V_TH_, confirming the suppression of electrically active traps. These results also indicate that vacuum annealing alone can contribute to a reduction in D_it_.

For quantitative comparison, D_it_ was determined via the high–low-frequency CV method using the following equation [28,29]:

(2) $$D_{it}=\frac{1}{qA}\left[{\left(\frac{1}{C_{LF}}-\frac{1}{C_{OX}}\right)}^{-1}-{\left(\frac{1}{C_{HF}}-\frac{1}{C_{OX}}\right)}^{-1}\right]$$

where D_it_ is the interface trap density, q is the elementary charge, A is the electrode area of the MOS capacitor, C_LF_ and C_HF_ are the capacitances measured at 5 kHz and 100 kHz, respectively, and C_OX_ is the oxide capacitance. Figure 3d shows the D_it_ values extracted at the V_TH_ of each device, where V_TH_ was 0.61 V for the device before annealing, 0.37 V for the vacuum-annealed device, and 0.16 V for the HPHA-treated device. The D_it_ value decreased from ~4.48 × 10^12^ eV^−1^cm^−2^ for the unannealed device to ~2.31 × 10^12^ eV^−1^cm^−2^ after vacuum annealing, and further to ~1.34 × 10^12^ eV^−1^cm^−2^ after HPHA.

This reduction in D_it_ after the annealing suggests that hydrogen effectively passivates the electrically active trap states in the ultrathin ZnO channel region (Table 2). The decrease in D_it_ is also consistent with the improved subthreshold swing and enhanced mobility observed in Table 1, highlighting the strong correlation between defect passivation and overall device performance.

To further investigate the dominant defect mechanisms affecting device performance, low-frequency noise measurements were carried out for three different device groups: unannealed, after vacuum annealing, and after HPHA. Low-frequency (1/f) noise—commonly referred to as flicker noise—constitutes the intrinsic noise source in oxide semiconductor TFTs [30,31,32,33]. Its power spectral density, S_ID_(f)∝1/f_γ_ with γ ≈ 1, arises from a carrier number fluctuation due to charge trapping/de-trapping at oxide/semiconductor interfaces (McWhorter model) [34,35] or from correlated mobility fluctuations caused by phonon or impurity scattering (Hooge model) [36,37]. In the McWhorter model [38], the normalized spectral density is expressed as

(3) $$\frac{S_{ID}\left(f\right)}{{I_{D}}^{2}}=\frac{q^{2}kTN_{t}}{WL{C_{ox}}^{2}f{\left(V_{GS}-V_{TH}\right)}^{2}}$$

where N_t_ is the oxide trap density, T the temperature, C_ox_ the gate–oxide capacitance per unit area, and W and L the channel width and length, respectively. In contrast, the Hooge mobility fluctuation model [38] gives

(4) $$\frac{S_{ID}\left(f\right)}{{I_{D}}^{2}}=\frac{q\alpha_{H}}{WLC_{ox}f\left(V_{GS}-V_{TH}\right)}$$

where α_H_ is the Hooge parameter. Using the differences in the noise characteristics, the differences in defect density can be compared.

**Figure 4:**
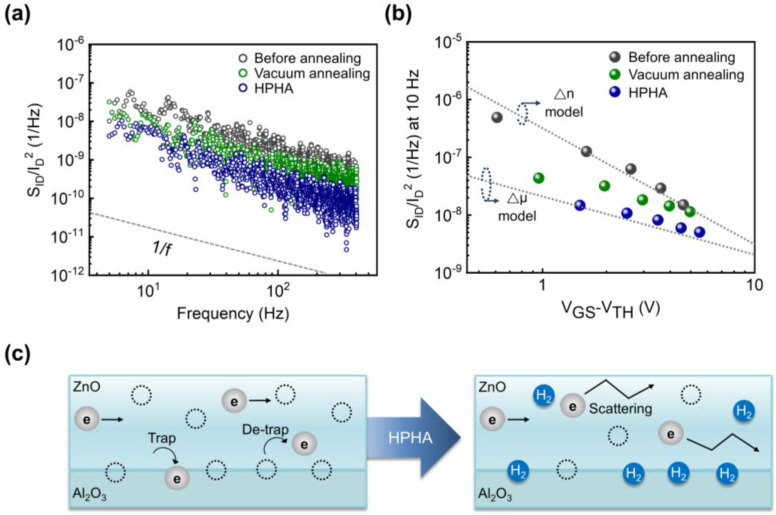
(**a**) Normalized current noise spectral density (S_ID_/I_D_^2^) as a function of frequency at V_GS_ = 6 V. All device groups show 1/f-type noise behavior. (**b**) S_ID_/I_D_^2^ at 10 Hz plotted versus V_GS_–V_TH_ of ZnO TFTs, illustrating a slope change from −2 (number fluctuation model) in the unannealed device to –1 (mobility fluctuation model) after HPHA. (**c**) Schematic of noise mechanism shift before (**left**) and after (**right**) HPHA. Before HPHA, number fluctuation by interfacial traps is dominant, and after HPHA, mobility fluctuation becomes dominant as oxygen-related traps are passivated by hydrogen.

Figure 4a shows the normalized current noise spectral density (S_ID_/I_D_^2^) of three device groups as a function of frequency at V_GS_ = 6 V. All devices exhibit 1/f-type behavior. Unannealed devices show relatively high noise levels, whereas vacuum annealing produces a modest decrease, consistent with partial trap removal. HPHA devices exhibit a substantial suppression of low-frequency noise.

From the slope of S_ID_/I_D_^2^ at 10 Hz versus the V_GS_–V_TH_ curve, the dominant mechanism of 1/f noise can be identified (Figure 4b). The slope for the unannealed devices follows approximately −2, characteristic of the Δn (number fluctuation) model, in which carriers tunnel in and out of traps located in the bulk and near the channel/dielectric interface. On the other hand, HPHA-treated devices follow a slope closer to −1, indicative of mobility fluctuation (Δμ) behavior described by the Hooge model, where carriers are scattered by lattice vibrations and residual impurities. Vacuum-annealed devices show intermediate behavior, indicating the presence of both mechanisms.

This transition from Δn to Δμ after HPHA reveals a fundamental change in the dominant noise mechanism: from trap-driven carrier number fluctuations in defective films to mobility fluctuations in trap-passivated channels. Figure 4c summarizes this mechanism shift, highlighting that hydrogen passivation of defects is the key to reducing trap-related noise.

## 4. Conclusions

In this work, we demonstrated that HPHA significantly improves the performance of ultrathin (3.6 nm) ZnO TFTs by effectively passivating both bulk and interface defect states. XPS analysis revealed a substantial reduction in oxygen vacancies and hydroxyl groups, while electrical characterization showed enhanced on-current, steeper subthreshold swing, and markedly reduced interface trap density. Most importantly, low-frequency noise analysis revealed a transition in the dominant noise mechanism from carrier number fluctuations (McWhorter model) in defective films to mobility fluctuations (Hooge model) in trap-passivated channels. These findings highlight the critical role of hydrogen in stabilizing ultrathin oxide semiconductors and provide physical insights as well as practical strategies for integrating low-dimensional oxide channels into future low-power and high-density electronic systems.

## Figures and Tables

**Table 1:** Extracted key electrical parameters of ZnO TFTs before annealing, after vacuum annealing, and after high-pressure hydrogen annealing (HPHA), enabling a direct comparison of device performance.

Device Type	On Current (μA)	Effective Mobility (cm^2^/V·s)	V_TH_ (V)	On/Off Ratio	S.S (mV/dec)
Before annealing	0.17 ± 0.02	1.4 ± 0.15	0.61 ± 0.10	1.43 × 10^8^	157
Vacuum annealing	0.35 ± 0.03	3.35 ± 0.11	0.37 ± 0.10	2.78 × 10^8^	133
HPHA	0.73± 0.1	5.31 ± 0.13	0.16 ± 0.05	1.32 × 10^9^	118

**Table 2:** Extracted capacitance and D_it_ value from high–low-frequency C–V method for the device before annealing, after vacuum annealing, and after high-pressure hydrogen annealing (HPHA).

Device Type	C_LF_ (pF)	C_HF_ (pF)	C_OX_ (pF)	D_it_ (eV^−1^cm^−2^)
Before annealing	5.43	3.07	12.82	4.48 × 10^12^
Vacuum annealing	3.60	2.34	9.20	2.31 × 10^12^
HPHA	3.49	2.68	10.63	1.34 × 10^12^

## Data Availability

The data presented in this study are available on request from the corresponding author.

## Abbreviations

The following abbreviations are used in this manuscript:

TFTs	Thin Film Transistors
HPHA	High Pressure Hydrogen Annealing
AFM	Atomic Force Microscopy
XPS	X-ray Photoelectron Spectroscopy
